# Diagnostic value and clinical laboratory associations of antibodies against recombinant ribosomal P0, P1 and P2 proteins and their native heterocomplex in a Caucasian cohort with systemic lupus erythematosus

**DOI:** 10.1186/ar3244

**Published:** 2011-02-10

**Authors:** Fidan Barkhudarova, Cornelia Dähnrich, Anke Rosemann, Udo Schneider, Winfried Stöcker, Gerd-Rüdiger Burmester, Karl Egerer, Wolfgang Schlumberger, Falk Hiepe, Robert Biesen

**Affiliations:** 1 Department of Rheumatology and Clinical Immunology, Charité Universitätsmedizin Berlin, Chariteplatz 1, Berlin D-10117, Germany; 2 EUROIMMUN Medizinische Labordiagnostika AG, Seekamp 31, Lübeck D-23560, Germany

## Abstract

**Introduction:**

In this study, we sought to determine the diagnostic value and clinical laboratory associations of autoantibodies against recombinant ribosomal P0, P1 and P2 proteins and their native heterocomplex in systemic lupus erythematosus (SLE).

**Methods:**

Autoantibodies against recombinant ribosomal P proteins (aRibP_R_0, aRibP_R_1 and aRibP_R_2) and antibodies against native ribosomal P heterocomplex (aRibP_N_H) were determined in sera from patients with SLE (*n *= 163), systemic sclerosis (*n *= 66), Sjögren's syndrome (*n *= 54), rheumatoid arthritis (*n *= 90) and healthy donors (*n *= 100) using enzyme-linked immunosorbent assay. Test results were correlated to medical records, including the American College of Rheumatology criteria, the Systemic Lupus Erythematosus Disease Activity Index 2000, laboratory data and medications of all SLE patients.

**Results:**

Sensitivities of 22.0% for aRibP_R_0, 14.9% for aRibP_R_2, 14.3% for aRibP_N_H and 10.7% for aRibP_R_1 were obtained at a specificity of 99%. The assay for aRibP_R_0 detection demonstrated the best performance in receiver-operating characteristics analysis, with aRibP_R_0 detectable in 10% of anti-Smith antibody and anti-double-stranded DNA-negative sera at a specificity of 100%. ARibP_R_0 positivity was associated with lymphocytopenia. ARibP_R_1^+ ^patients had significantly higher γ-glutamyl transpeptidase (GGT) levels than their aRibP_R_1^- ^counterparts. No specific damage occurred in aRibP^+ ^lupus patients compared with a group of age-, sex- and nephritis-matched aRibP^- ^lupus patients within 3 years.

**Conclusions:**

The determination of antibodies against ribosomal P proteins improves the diagnosis of SLE and should therefore be implemented in upcoming criteria for the diagnosis or classification of SLE. High titers of aRibP_R_0 can be associated with lymphocytopenia, and high titers of aRibP_R_1 can be associated with elevated GGT levels. So far, there is no evidence for a prognostic value of aRibPs for damage.

## Introduction

Systemic lupus erythematosus (SLE) is a chronic, multifaceted rheumatic disease which is characterised by the generation of autoantibodies predominantly directed against nuclear proteins and nucleic acids [1,2]. However, antibodies against cytoplasmatic antigens such as those binding to ribosomal P proteins (aRibPs) have been reported to be specific for SLE as well [2,3]. In contrast to anti-Smith (anti-Sm) and anti-double-stranded DNA (anti-dsDNA) antibodies, anti-ribosomal P protein antibodies are not included in the current American College of Rheumatology (ACR) classification criteria for SLE [4,5].

The human ribosomal phosphoproteins P0 (38 kDa), P1 (19 kDa) and P2 (17 kDa) are located within the 60S ribosomal subunit, forming a pentameric complex consisting of a P0 anchor and two P1/P2 heterodimers [3]. The subunits of that pentamer have a common immunodominant epitope at the carboxyl terminus [6], which can lead to cross-reactions of anti-ribosomal P antibodies with P0, P1 and P2 units. P proteins can also exist as ribosome-free P0, P1 and P2 forms in the cytoplasm [6,7]. Notably, the P0-like protein is also detectable in the plasma membranes of hepatocytes, lymphocytes and other cells [8-11].

The prevalence of anti-ribosomal antibodies depends on the disease activity, the patient's ethnicity and the antigens used in detection systems [12-14]. There are reports about clinical associations of anti-ribosomal protein antibodies with short disease duration [15], rash [16,17], lymphocytopenia [18] and lupus hepatitis [11,19-23]. Ohira *et al. *[22] showed that patients with lupus hepatitis have significantly higher and more frequent levels of antibodies against recombinant ribosomal P0 protein (aRibP_R_0) than patients with autoimmune hepatitis. There are also contradictory reports of patients with juvenile onset SLE [24-27], neuropsychiatric SLE [3,28,29], lupus nephritis class V [3,27,30], high disease activity [15,16,26,31] and low levels of complement component 3 (C3) or complement component 4 (C4) [16,17,22,32].

A comparative investigation of the clinical laboratory associations of antibodies against recombinant ribosomal P0, P1 and P2 proteins (aRibP_R_0, aRibP_R_1 and aRibP_R_2) has never been conducted. Thus, the purpose of the present work was to determine the diagnostic value of antibodies against native ribosomal P heterocomplex (aRibP_N_H), aRibP_R_0, aRibP_R_1 and aRibP_R_2 for SLE and to analyse their associations with disease features and future damage.

## Materials and methods

### Study participants

Altogether 479 serum samples were obtained from the following groups: (1) patients with SLE (*n *= 163), who fulfilled the American College of Rheumatology (ACR) 1982 revised criteria for the classification of SLE [4], (2) patients with systemic sclerosis (SSc, *n *= 66) who met the ACR 1980 criteria for scleroderma [33], (3) patients with primary Sjögren's syndrome (pSS, *n *= 54) who fulfilled the preliminary European League Against Rheumatism criteria of Vitali *et al. *[34], (4) patients with rheumatoid arthritis (RA, *n *= 90) who met the ACR 1987 revised criteria for the classification of rheumatoid arthritis [35] and (5) healthy donors (HD, *n *= 100).

Disease activity of SLE patients was defined based on the Systemic Lupus Erythematosus Disease Activity Index 2000 (SLEDAI 2000) [36-38] in 101 patients: 6 of them had no activity (SLEDAI score 0), 35 were mildly active (0 < SLEDAI ≤ 5), 41 had moderate disease activity (5 < SLEDAI ≤ 10), 14 were highly active (10 < SLEDAI ≤ 20), and 5 had very high activity (SLEDAI > 20). Juvenile onset was diagnosed when the age at diagnosis was 18 years or younger according to the Pediatric Rheumatology International Trials Organization [39]. Twenty-four (14.7%) patients with juvenile onset SLE and 139 (85.3%) patients with adult onset SLE were studied. Disease damage was measured according to the criteria of the Systemic Lupus International Collaborative Clinics (SLICC) [40,41] and the weighted damage score (WDS) [40]. All patients were recruited from the outpatient and inpatient facilities of the Department of Rheumatology and Clinical Immunology, Charité University Hospital, Berlin, Germany. The Ethics Committee of the Medical Faculty of Charité University Hospital approved the study, and written informed consent was obtained from all subjects. Sera from healthy donors were used in cooperation with University of Lübeck, Germany. Written informed consent was obtained from all healthy subjects.

### Measurement of antibodies

Microtiter plates (Nunc, Roskilde, Denmark) were coated with 1 μg/ml full-length recombinant ribosomal protein P0, P1 or P2 expressed in insect cells (DIARECT, Freiburg, Germany). Sera diluted 1:201 in phosphate-buffered saline (PBS) and 0.1% (wt/vol) casein were added and allowed to react for 30 minutes, followed by three washing cycles with PBS 0.05% (vol/vol) and Tween 20. For detection of bound antibodies, the plates were incubated with antihuman immunoglobulin (IgG) peroxidase conjugate (EUROIMMUN, Lübeck, Germany) for 30 minutes, washed three times and allowed to react with tetramethylbenzidine (EUROIMMUN) for 15 minutes. After addition of acidic stopping solution (EUROIMMUN), the optical density (OD) was read at 450 nm using an automated spectrophotometer (Spectra Mini; Tecan, Crailsheim, Germany). All steps were performed at room temperature. A highly positive index patient serum was used to generate a standard curve consisting of three calibrators (2, 20 and 200 relative units (RU)/ml). Relative units per milliliter were calculated for all samples using this three-point standard curve. The analytical reproducibility of all aRibP assays was evaluated by repeated testing of two serum samples (10 determinations each) in the same run, giving intraassay coefficients of variation (CV) of 2.4% (aRibP_R_0), 2.1% (aRibP_R_1) and 2.7% (aRibP_R_2), respectively. Relationships between sensitivity and specificity at different cutoff values were examined for all assays by receiver-operating characteristics (ROC) curve analyses, allowing also for the determination of test characteristics at predefined specificities.

The anti-RibP_N_H enzyme-linked immunosorbent assay (ELISA) (IgG, CV 2.6%), anti-Sm ELISA, anti-dsDNA radioimmunoassay (RIA) (Farr assay) and anti-dsDNA ELISA are commercially available assays from EUROIMMIUN and were performed following the manufacturer's instructions.

### Statistical analysis

Statistical analyses were performed using GraphPad Prism 5 software (GraphPad Software, La Jolla, CA, USA). The diagnostic significance of antiribosomal proteins N, P0, P1 and P2 antibodies was assessed and areas under the curve (AUCs) were created using ROC analysis. To determine associations, the Mann-Whitney *U *test (for comparing medians between groups; MWT), Fisher's exact test (FET) and Spearman's rank test (SRT) were used. Two-tailed *t*-tests were used throughout with an α set at 0.05.

## Results

### Reactivity and diagnostic significance of antiribosomal proteins N, P0, P1 and P2 antibodies

**Figure 1:**
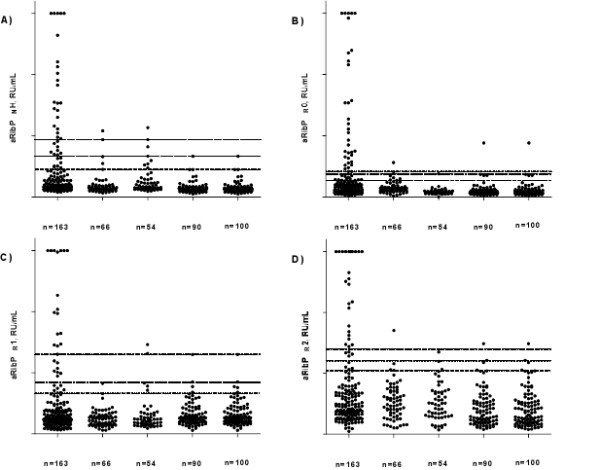
**Graphs showing levels of antiribosomal P protein antibodies in SLE, other rheumatic diseases and healthy donors**. Autoantibodies directed against **(a) **native ribosomal P heterocomplex (aRibP_N_H), **(b) **recombinant ribosomal P0 protein (aRibP_R_0), **(c) **recombinant ribosomal P1 (aRibP_R_1) and **(d) **recombinant ribosomal P2 protein (aRibP_R_2) were measured using enzyme-linked immunosorbent assay. Dotted lines represent the distinct cut-offs based on ROC curve analysis at specificities of 95% (dotted line), 98% (broken line) and 99% (dotted and broken line). Values >30 RU/ml were set to 30 RU/ml for clearer arrangement of the figures. SLE, systemic lupus erythematosus; SSc, systemic sclerosis; pSS, primary Sjögren's syndrome; RA, rheumatoid arthritis; HD, healthy donors. RU, relative units.

Antibodies against ribosomal P_N_H, P_R_0, P_R_1 and P_R_2 proteins (Figure 1), Sm and dsDNA (ELISA and Farr assays) were measured in sera from 163 SLE patients, 210 disease controls and 100 healthy donors to define and compare the sensitivity and specificity in ROC curve analysis (Table 1). For aRibP_N_H, a sensitivity of 5.5% and a specificity of 100% were calculated using the manufacturer's cutoff (20 RU/ml). At a predefined specificity of 98% among 210 patients with other rheumatic diseases (SSc, pSS and RA), only five (2.4%), four (1.9%), four (1.9%) and four (1.9%) had elevated aRibP_N_H, aRibP_R_0, aRibP_R_1 and aRibP_R_2 titers, respectively. At the same specificity among 100 healthy donors, only zero (0%), one (1.0%), two (2.0%) and two (2.0%) patients had high titers of aRibP_N_H, aRibP_R_0, aRibP_R_1 and aRibP_R_2. Among antiribosomal P protein antibodies, aRibP_R_0 had the highest performance with regard to criteria such as AUC and maximum sum of sensitivity and specificity, followed by aRibP_N_H (Table 1). All test criteria of aRibP_R_0 were inferior to those of the anti-dsDNA ELISA or the Farr assay, but were almost equal to those of the anti-Sm ELISA.

**Table 1:** Test values of antiribosomal P_N_H, P_R_0, P_R_1 and P_R_2 antibodies calculated in receiver-operating characteristics analysis^a^

Statistics	**aRibP**_**N**_**H**	**aRibP**_**R**_**0**	**aRibP**_**R**_**1**	**aRibP**_**R**_**2**	Anti-Sm	Anti-dsDNA Farr assay	Anti-dsDNA ELISA
Area under curve	0.7014	0.7368^b^	0.5811	0.6220	0.6791	0.8463	0.8621
95% CI	0.65 to 0.75	0.69 to 0.79	0.52 to 0.64	0.57 to 0.67	0.62 to 0.74	0.80 to 0.89	0.82 to 0.90
*P *value	<0.0001	<0.0001	0.0021	<0.0001	<0.0001	<0.0001	<0.0001
Sensitivity at 95% specificity cutoff	24.4% (4.5)	29.2% (2.7)^b^	20.4% (6.6)	20.2% (10.5)	38.7% (2.0)	61.4% (5.4)	53.9% (73.9)
Sensitivity at 98% specificity cutoff	19.1% (6.7)	22.0% (3.7)^b^	16.1% (8.4)	17.9% (12.1)	33.7% (2.4)	56.4% (6.5)	42.9% (105.8)
Sensitivity at 99% specificity cutoff	14.3% (9.4)	22.0% (4.2)^b^	10.7% (13.0)	14.9% (13.9)	19.6% (4.8)	55.8% (6.8)	37.4% (151.0)
Sensitivity at 100% specificity cutoff	11.9 (11.5)^b^	11.3% (9.1)	8.9% (14.7)	11.3% (17.4)	12.3% (7.9)	49.1% (9.0)	31.3% (169.0)
Maximum sum of specificity and sensivity	133.2%	140.7%^b^	118.2%	117.9%	138.9%	161.8%	160.8%

^a^aRibP_N_H, antibody against native ribosomal P heterocomplex; aRibP_R_0, antibody against recombinant ribosomal P0 protein; aRibP_R_1, antibody against recombinant ribosomal P1 protein; aRibP_R_2, antibody against recombinant ribosomal P2 protein; anti-Sm, anti-Smith antibody; anti-dsDNA, anti-double-stranded DNA antibody; ELISA, enzyme-linked immunosorbent assay; 95% CI, 95% confidence interval; ^b^highest values of sensitivity, area under the curve and lowest cutoff values (in parentheses) among autoantibodies against ribosomal P protein (aRibP).

### Patients negative for aRibP_N_H but positive for aRibP_R_P0-2

Although the native heterocomplex of ribosomal P contains all immunological domains of the subunits P0, P1 and P2, there were considerable differences in the cutoffs and in sensitivities for the detection of aRibP_N_H, aRibP_R_0, aRibP_R_1 and aRibP_R_2 (Table 1), with outstanding results for aRibP_R_0.

**Figure 2:**
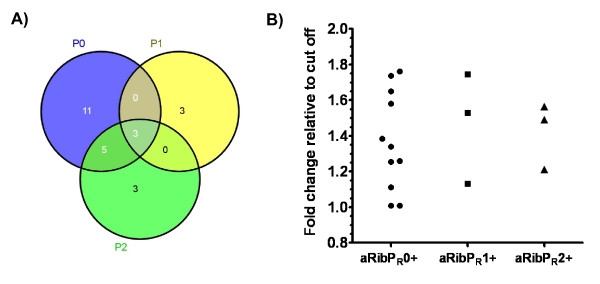
**Frequencies of aRibP_R_0, aRibP_R_1 and aRibP_R_2 in aRibP_N_H-negative lupus patients**. **(a) **Results according to specificity of 99% are shown in a Venn diagram. **(b) **Corresponding to Figure 2a, those sera were selected that were exclusively positive for aRibP_R_0, aRibP_R_1 or aRibP_R_2 among aRibP_N_H-negative SLE patients. To further show exact and comparable signal intensities, fold change indices in relation to the given cutoffs of each recombinant aRibP protein (see also Table 1) were calculated. ARibP_N_H, antibodies against native ribosomal P heterocomplex; aRibP_R_0, antibodies against recombinant ribosomal P0 protein; aRibP_R_1, antibodies against recombinant ribosomal P1 protein; aRibP_R_2, antibodies against recombinant ribosomal P2 protein; aRibPs, anti-ribosomal P protein antibodies.

Thus, we further investigated whether there were patients negative for aRibP_N_H but positive for aRibP_R_0, aRibP_R_1 or aRibP_R_2 (Figure 2). Sera fulfilling these criteria would point out that there are some epitopes of ribosomal P proteins that are not accessible to autoantibodies because of the spatial conformation of the native heterocomplex.

At 99% specificity, among 141 aRibP_N_H^- ^patients there were 19 (13.5%) positive for aRibP_R_0, six (4.3%) positive for aRibP_R_1 and 11 (7.8%) positive for aRibP_R_2. Some of those sera were further exclusively positive for one of the recombinant aRibPs and showed an increased titer up to twofold of the corresponding cutoff (Figure 2b).

### Diagnostic value of anti-ribosomal P protein antibodies in SLE

**Figure 3:**
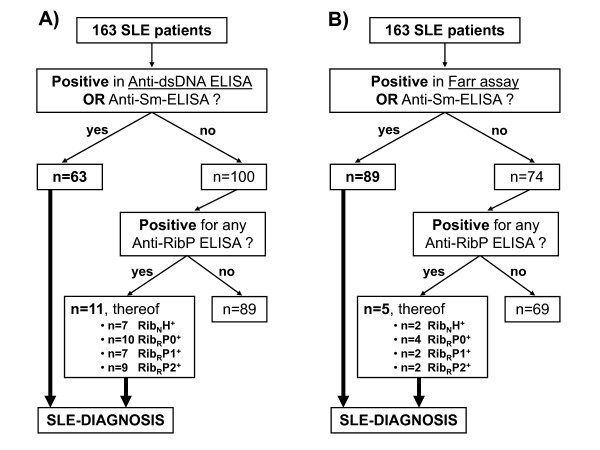
**Additional diagnostic benefit of antiribosomal P protein antibodies in lupus patients**. Both flow charts aim to demonstrate the additional diagnostic value of antiribosomal P protein antibodies (anti-RibP) in the absence of anti-double-stranded DNA (anti-dsDNA) and anti-Smith (anti-Sm) antibodies for lupus diagnostics. The cutoffs of all test systems were set to ensure an absolute specificity of 100% (see Table 1 for cutoffs). Anti-dsDNA and anti-Sm antibodies were selected because they are highly specific for systemic lupus erythematosus (SLE) (instead of, for example, anti-phospholipid antibodies) and are part of American College of Rheumatology classification criteria for SLE [4]. Flowcharts differ only in the test system used for the detection of anti-dsDNA antibodies. **(A) **Anti-dsDNA enzyme-linked immunosorbent assay (ELISA) and **(B) **Farr assay. RibP_N_H^+^, native ribosomal P heterocomplex-positive; RibP_R_0^+^, recombinant ribosomal P0 protein-positive; RibP_R_1^+^, recombinant ribosomal P1 protein-positive; RibP_R_2^+^, recombinant ribosomal P2 protein-positive.

To investigate the auxiliary diagnostic value of antiribosomal P protein antibodies in SLE, we searched for patients who were negative for antibodies against dsDNA and Sm, but positive for aRibP_N_H, aRibP_R_0, aRibP_R_1 or aRibP_R_2 at a specificity of 100% (Figure 3). This analysis was performed twice, taking either the results of the anti-dsDNA ELISA (Figure 3a) or those of the Farr assay (Figure 3b).

Among 163 SLE patients, there were 11 (6.7%) individuals who could be diagnosed only by detection of aRibPs, while 63 (38.7%) patients were regularly diagnosed by the presence of anti-dsDNA or anti-Sm antibodies. Considering the excellent Farr assay, these relations adjusted to 89 (54.6%) individuals with regular diagnosis and five (3.1%) individuals with additional diagnosis only by the presence of aRibP.

### Comparison of disease features in aRibP^+ ^vs. aRibP^- ^SLE patients

To determine the special characteristics of lupus patients with elevated aRibPs, we compared medical records, including ACR criteria, SLEDAI 2000 items and laboratory parameters, including autoantibodies, immunosuppressants and antimalarials, with those of aRibP^- ^lupus patients. All clinical laboratory results and detailed demographic information about the study cohort are shown in Table 2.

**Table 2:** Comparison of the frequency: demographical and clinical data in aRibP-positive and negative SLE patients^a^

		**aRibP**_**N**_**H**			**aRibP**_**R**_**0**			**aRibP**_**R**_**1**			**aRibP**_**R**_**2**		
					
Clinical data	All patients (*n *= 163)	Positive (*n *= 30)	Negative (*n *= 133)	* P *value	Positive (*n *= 34)	Negative (*n *= 129)	* P *value	Positive (*n *= 24)	Negative (*n *= 139)	* P *value	Positive (*n *= 28)	Negative (*n *= 135)	* P *value
Demographics													
Age in years,^b ^median	37.0	36.5	37.0	0.317	35.0	37.0	0.081	35.0	37.0	0.326	37.0	37.0	0.467
Age at onset,^c ^<18 years, %	19.7	15.6	13.9	1.000	24.3	16.8	0.338	22.2	17.7	0.592	26.7	16.7	0.203
Females,^c ^%	89.8	84.4	91.2	0.324	89.2	90	1.000	96.3	88.7	0.314	90	89.9	1.000
SLE duration in months,^b ^median	108.0	97.0	109.0	0.716	69.0	109.0	0.118	62.0	109.0	0.147	96.0	107.0	0.756
ACR criteria	*n *= 163	*n *= 30	*n *= 133		*n *= 34	*n *= 129		*n *= 24	*n *= 139		*n *= 28	*n *= 135	
Number of ACR criteria,^b ^median	6.00	**7.00**^**d**^	**6.00**^**d**^	**0.031**^**d**^	7.00	6.00	0.059	6.50	6.00	0.236	7.00	6.00	0.076
Malar rash,^c ^%	66.2	73.3	64.6	0.402	70.6	65.1	0.684	70.8	65.5	0.815	71.4	65.2	0.662
Discoid rash,^c ^%	12.3	16.7	11.3	0.536	14.7	11.6	0.571	16.7	11.5	0.501	25.0	9.63	0.0504
Photosensitivity,^c ^%	46.6	**63.3**^**d**^	**42.9**^**d**^	**0.046**^**d**^	58.8	43.4	0.125	62.5	43.9	0.121	53.6	45.2	0.533
Oral ulcers,^c ^%	18.4	23.3	17.3	0.441	23.5	17.1	0.456	12.5	19.4	0.573	17.9	18.5	1.000
Arthritis,^c ^%	84.1	90.0	82.7	0.417	94.1	81.4	0.111	91.7	82.7	0.373	96.4	81.5	0,0506
Serositis,^c ^%	44.2	36.7	45.9	0.419	38.2	45.7	0.561	41.7	44.6	0.828	39.3	45.2	0.677
Renal disorder,^c ^%	42.9	46.7	42.2	0.686	44.1	42.6	1.000	37.5	43.9	0.658	39.3	43.7	0.834
Epilepsy or psychosis,^c ^%	12.9	20.0	11.3	0.228	14.7	12.4	0.774	25.0	10.8	0.091	21.4	11.1	0.209
Hematologic,^c ^%	63.8	70.0	62.4	0.530	67.6	62.8	0.690	58.3	64.7	0.646	67.9	62.9	0.672
Immune disorder and ANA, %	100	100	100	-	100	100	-	100	100	-	100	100	-
SLEDAI	*n *= 101	*n *= 17	*n *= 84		*n *= 22	*n *= 79		*n *= 17	*n *= 84		*n *= 17	*n *= 84	
SLEDAI,^b ^median	6.00	6.00	6.00	0.517	6.00	6.00	0.886	6.00	6.00	0.915	6.00	6.00	0.256
Vasculitis,^c ^%	7.92	5.89	8.33	1.000	4.55	8.86	0.682	11.8	7.14	0.621	5.89	8.33	1.000
Arthritis,^c ^%	33.7	23.5	35.7	0.408	22.7	36.7	0.309	**11.8**^**d**^	**38.1**^**d**^	**0.048**^**d**^	**11.8**^**d**^	**38.1**^**d**^	**0.048**^**d**^
Hematuria,^c ^%	6.93	11.8	5.95	0.335	4.55	7.59	1.000	0.00	9.52	0.346	0.00	9.41	0.346
Proteinuria,^c ^%	9.90	17.6	8.33	0.366	9.09	10.1	1.000	5.88	10.7	1.000	5.88	10.7	1.000
Exanthema,^c ^%	32.7	11.8	36.9	0.0503	27.3	34.2	0.615	23.5	34.5	0.572	23.5	34.5	0.572
Alopecia,^c ^%	16.8	23.5	15.5	0.478	27.3	13.9	0.195	17.6	16.7	1.000	17.6	16.7	1.000
Pericarditis,^c ^%	6.93	5.88	7.14	1.000	9.09	6.33	0.645	11.8	5.95	0.335	5.88	7.14	1.000
Complement deficiency,^c ^%	57.4	64.7	55.9	0.597	68.2	51.2	0.331	58.8	57.1	1.000	64.7	55.9	0.597
Fever,^c ^%	3.96	0.00	4.67	1.000	4.55	3.79	1.000	0.00	4.76	1.000	0.00	4.76	1.000
Drugs													
Prednisolone,^c ^%	89.9	93.1	89.2	0.738	90.9	89.7	1.000	86.9	90.4	0.706	88.9	90.3	0.734
Azathioprine,^c ^%	30.8	27.6	31.5	0.825	36.4	29.4	0.526	17.4	33.1	0.151	29.6	31.1	1.000
Cyclophosphamide,^c ^%	8.81	6.89	9.23	1.000	12.1	7.94	0.491	18.8	7.75	0.155	11.1	8.33	0.708
Mycophenolate mofetil,^c ^%	20.5	20.7	19.7	1.000	15.2	22.2	0.474	30.4	19.1	0.265	22.2	20.5	0.799
Methotrexate,^c ^%	3.04	3.23	3.01	1.000	8.33	1.56	0.071	7.69	2.17	0.179	3.45	2.96	1.000
Hydroxychloroquine sulfate,^c ^%	35.4	50.0	32.3	0.085	36.4	35.2	1.000	30.4	36.3	0.645	37.0	35.1	0.829
Laboratory													
Leukocytes,^b ^median	6.50	7.03	6.40	0.539	7.00	6.30	0.136	6.58	6.40	0.479	6.50	6.49	0.933
Lymphocytes,^b ^median	0.87	0.67	0.87	0.164	**0.63**^**d**^	**0.92**^**d**^	**0.036**^**d**^	0.91	0.86	0.957	0.70	0.93	0.076
Monocytes,^b ^median	0.35	0.34	0.35	0.769	0.33	0.35	0.584	0.48	0.35	0.301	0.31	0.35	0.540
ALT,^b,e ^median	19.4	14.4	20.0	0.501	22.0	19.2	0.824	20.7	19.0	0.661	14.7	20.0	0.547
AST,^b,e ^median	25.0	28.0	24.9	0.212	25.5	25.0	0.953	27.5	24.9	0.129	25.0	25.0	0.568
GGT,^b,e ^median	23.0	26.0	21.6	0.278	24.0	21.1	0.423	**29.0**^**d**^	**21.0**^**d**^	**0.047**^**d**^	29.0	21.1	0.108
Low C3,^c ^%	47.6	**65.5**^**d**^	**43.2**^**d**^	**0.038**^**d**^	58.1	44.8	0.227	54.2	46.3	0.511	61.5	44.6	0.134
Low C4,^c ^%	34.7	37.9	33.9	0.670	38.7	33.6	0.672	37.5	34.1	0.816	38.4	33.9	0.656
CRP,^b ^median	0.38	0.30	0.40	0.771	0.28	0.42	0.454	0.25	0.42	0.379	26.0	25.9	0.803
ESR,^b ^median	24.0	25.0	24.0	0.616	22.0	25.0	0.740	21.5	25.0	0.572	23.0	24.5	0.819
Autoantibodies													
High anti-Sm,^c ^%	33.7	**63.3**^**d**^	**27.1**^**d**^	**4 × 10**^**-3d**^	**70.6**^**d**^	**24.2**^**d**^	**<10**^**-4d**^	**66.7**^**d**^	**28.1**^**d**^	**7 × 10**^**-3d**^	**60.7**^**d**^	**28.1**^**d**^	**0.002**^**d**^
Increased anti- dsDNA in ELISA,^c ^%	42.3	**70.0**^**d**^	**36.8**^**d**^	**0.002**^**d**^	**67.8**^**d**^	**36.4**^**d**^	**0.002**^**d**^	**75.0**^**d**^	**37.4**^**d**^	**7 × 10**^**-3d**^	**60.7**^**d**^	**39.3**^**d**^	**0.058**^**d**^
Increased anti- dsDNA in RIA,^c ^%	56.4	**76.7**^**d**^	**51.9**^**d**^	**0.015**^**d**^	**79.4**^**d**^	**50.4**^**d**^	**0.003**^**d**^	**83.3**^**d**^	**51.8**^**d**^	**0.004**^**d**^	**78.6**^**d**^	**51.9**^**d**^	**0.012**^**d**^
Increased anti- U_1_-RNP in anamnesis,^c ^%	28.9	**59.1**^**d**^	**21.7**^**d**^	**0.001**^**d**^	**50.0**^**d**^	**23.3**^**d**^	**0.021**^**d**^	**62.5**^**d**^	**23.5**^**d**^	**0.003**^**d**^	**54.5**^**d**^	**22.7**^**d**^	**0.007**^**d**^
Increased anti- nucleosomes,^c ^%	50.9	60.0	48.9	0.315	64.7	47.3	0.084	62.5	48.9	0.271	**70.4**^**d**^	**47.1**^**d**^	**0.035**^**d**^
Increased anti- Ro/SS-A,^c ^%	40.5	50.0	38.3	0.304	41.2	40.3	1.000	37.5	41.0	0.824	39.3	40.7	1.000
Increased anti- La/SS-B,^c ^%	12.3	20.0	10.5	0.213	8.82	13.2	0.769	**0.00**^**d**^	**14.4**^**d**^	**0.046**^**d**^	3.57	14.1	0.203

^a^SLE, systemic lupus erythematosus; ACR, American College of Rheumatology; ANA, antinuclear antibody; SLEDAI, Systemic Lupus Erythematosus Disease Activity Index; ALT, alanine aminotransferase; AST, aspartate aminotransferase; GGT, γ-glutamyl transpeptidase; CRP, C-reactive protein; ESR, erythrocyte sedimentation rate; ELISA, enzyme-linked immunosorbent assay; RIA, radioimmunoassay; anti-Sm, anti-Smith antibody; anti-dsDNA, anti-double-stranded DNA antibody; Ro/SS-A, anti- Sjögren's syndrome antigen A; La/SS-B, anti- Sjögren's syndrome antigen B; U1-RNP, U_1_-ribonucleoprotein; aRibP, anti-ribosomal P protein antibody; aRibP_N_H, antibodies against native ribosomal P heterocomplex; aRibP_R_0, antibodies against recombinant ribosomal P0 protein; aRibP_R_1, antibodies against recombinant ribosomal P1 protein; aRibP_R_2, antibodies against recombinant ribosomal P2 protein. All used autoantibody cutoffs were read out of receiver-operating characteristics analysis at a specificity of 98% (see Table 1); ^b^*P *values were calculated using the Mann-Whitney *U *test; ^c^*P *values were calculated using Fisher's exact test; ^**d**^**statistically significant findings, highlighted in bold; **^e^GGT values of men and from other laboratories have been standardized on cutoffs of GGT for women at the Charité University Hospital.

ARibP_N_H^+ ^patients fulfilled significantly more ACR criteria and more often had photosensitivity. Moreover, the frequency of patients with decreased C3 levels was higher among aRibP_N_H^+ ^patients. Lymphocytopenia was associated with the presence of aRibP_R_0, and a higher γ-glutamyl transpeptidase (GGT) level was found in aRibP_R_1^+ ^patients. Anti-Sm, anti-dsDNA and anti-U_1_-ribonucleoprotein (anti-U_1_-RNP) antibodies were much more frequent in all aRibP^+ ^patients.

### Comparison of disease damage in aRibP^+ ^vs. aRibP^- ^SLE patients

To study the prognostic role of ribosomal P protein antibodies, SLICC scores and WDS were assessed in aRibP^+ ^patients and in an age-, sex- and nephritis-matched group of aRibP^- ^patients at the time of blood sampling and 3 years later. Changes in damage scores (ΔSLICC, ΔWDS) were calculated, and both groups were separately compared. Damage scores from 41 of all 58 aRibP^+ ^patients were completely assessable at the time of blood sampling and 3 years later. There were 22 aRibP_N_H^+^, 27 aRibP_R_0^+^, 18 aRibP_R_1^+ ^and 23 aRibP_R_2^+ ^patients. SLICC and WDS correlated significantly with disease duration and the ages of patients, but not with ACR scores or with anti-dsDNA, anti-Sm or any antiribosomal P protein antibodies. Total disease damage and damage to every organ system separately was not significantly higher in aRibP^+ ^patients than in their aRibP^- ^counterparts within these 3 years. Thus, we found no prognostic role for aRibP.

## Discussion

In this study, the diagnostic potential, clinical laboratory associations and correlations with disease damage of antibodies directed against the native ribosomal heterocomplex and its recombinantly produced constituents P0, P1 and P2 were investigated. ARibP_R_0 revealed the best diagnostic performance among all aRibP types and offered the most diagnostic benefit among sera negative for anti-dsDNA and anti-Sm antibodies. Furthermore, aRibP_R_0^+ ^lupus patients had significantly lower lymphocyte counts than their aRibP_R_0^- ^counterparts. Finally, no prognostic relevance was found for any of the aRibPs during a 3-year period.

Our results concerning the prevalence and high specificity of aRibPs for SLE agree with data described before [3,42]. We further found sensitivities of P_R_0 > P_N_H > P_R_2 > P_R_1 at specificities of 98% to 99% and P_N_H > P_R_0 = P_R_2 > P_R_1 at a specificity of 100% in a cohort of 163 lupus patients. This is in contrast to another study where sensitivities of P_R_2 = P_R_1 = P_R_0 were determined at a specificity of 100% in a cohort of 50 SLE patients [13]. Different detection systems and patient cohorts might have contributed to these divergent observations. Since all three subunits of aRibPs share the carboxyl-terminal epitope, it is of interest to note that an ELISA (referred as anti-C22 ELISA) detecting antibodies against this shared epitope reached the same sensitivity of 22% at a specificity of nearly 99% as aRib P_R_0 in our Berlin patient cohort [43].

We have additionally demonstrated that negativity of aRibP_N_H does not automatically imply negativity of antibodies against its subunits, especially those against ribosomal P0. This could be due to immunologically relevant epitopes that are freely accessible using RibP_R_0 alone, but not within the spatial conformation of the native heterocomplex. A biological reason for the higher frequency of aRibP_R_0 might be the disposability of ribosomal P0-like protein in the cell membranes of many cells, which could contribute to an increased immunogenicity [8-11].

Among the vast quantity of antibodies that are detectable in SLE, antibodies against dsDNA and Sm are highly specific and therefore most useful for the verification of the diagnosis. However, aRibPs are also discussed as a diagnostic criterion. Therefore, we asked whether aRibPs provide additional diagnostic benefit in direct comparison to anti-dsDNA and anti-Sm antibodies. Exactly 10% of sera negative in the anti-Sm and anti-dsDNA ELISAs were positive for aRibP_R_0 at a specificity of 100%. Even the comparison including the Farr assay revealed that 5.4% of all anti-Sm ELISA and anti-dsDNA RIA (Farr assay) negative sera were positive for aRibP_R_0 at 100% specificity. Thus, laboratories using less sensitive assays seem to benefit more from testing for aRibP in suspected cases of SLE. However, to be sure, all patients with suspected diagnosis of SLE should be tested for aRibP. Finally, we conclude that the determination of antibodies against ribosomal P proteins, especially those against P0, would improve the classification and diagnosis of SLE.

By comparing disease features of lupus patients with elevated aRibPs to their seronegative counterparts, we could not confirm an association of aRibP positivity with lupus nephritis, short disease duration, high disease activity or juvenile onset. These results might be influenced by the Caucasian ethnicity of the study cohort and differences of the test systems. Cases of neuropsychiatric lupus [28,29] and subtypes of lupus nephritis were not recorded in our study.

The most striking association of aRibPs with disease features was that aRibP_R_0^+ ^lupus patients had significantly lower lymphocytes than aRibP_R_0^- ^lupus patients. Interestingly, a P0-like protein is also detectable in the plasma membranes of different cells, including lymphocytes [11]. Further, the aRibPs are able to bind and penetrate T-cell lines [44,45], and especially aRibP_R_0 can induce apoptosis in Jurkat T-cells [46]. In that context, our data confirm the thesis of Sun *et al. *[46] that aRibP_R_0 contributes in a clinically relevant manner to lymphocytopenia in SLE. Thus, clinicians should keep aRibP_R_0 in mind as one differential diagnosis for lymphocytopenia in SLE, along with viral status, drug side effects, hematologic malignancies and other factors.

Another remarkable, significant clinical laboratory association was that aRibP_R_1^+ ^patients had an elevated GGT value. The participation of aRibPs in liver pathology of SLE was previously reported in cell cultures [9,11,46] and in case reports [19-21]. However, aRibP_R_0 were most frequently in focus because of their membrane-bound isoform [8-11]. As such, in a study of 61 Japanese patients [22], no significant association was found between aRibP_R_0 and liver enzymes alanine aminotransferase or aspartate aminotransferase, but the GGT level was not assessed. The correlation shown here between GGT and aRibP_R_1 indicates a possible association of aRibP with lupus hepatitis. However, we do not have a clear definition of lupus hepatitis, and it is hard to rule out other causes, such as nutrition, drugs and other autoimmune hepatitis forms. Longitudinal analysis of aRibPs with liver function tests, including GGT in parallel, might reveal this association best.

Up to now, accepted prognostic factors in SLE have only been lupus nephritis and neuropsychiatric SLE. No prognostic laboratory parameter is known. In this study, we investigated whether aRibP^+ ^lupus patients would develop more or specific disease damage measured by SLICC or WDS after 3 years than their aRibP^- ^counterparts. However, no significant correlations with any of the antiribosomal P protein antibodies could be found over a 3-year period. Conclusively, we first show that aRibPs are not a prognostic parameter for damage in SLE. Further study with more patients and over longer observation time frames could strengthen this result.

## Conclusions

In summary, antiribosomal P protein antibodies are very specific for SLE, can also be found in patients with negative anti-dsDNA and anti-Sm antibodies and therefore have to be discussed in the upcoming classification and diagnostic criteria for SLE. Among all four investigated aRibPs, aRibP_R_0 was the most abundant and should be used for the diagnosis of SLE. High aRibP_R_0 titers can be associated with lymphocytopenia, and high aRibP_R_1 titers can be associated with an elevated GGT level. A prognostic role of antiribosomal P protein antibodies is unlikely.

## Abbreviations

aRibP_N_H: antibodies against native ribosomal P heterocomplex; aRibP_R_0: antibodies against recombinant ribosomal P0 protein; aRibP_R_1: antibodies against recombinant ribosomal P1 protein; aRibP_R_2: antibodies against recombinant ribosomal P2 protein; aRibPs: anti-ribosomal P protein antibodies.

## Competing interests

RB was employed from August 2006 until March 2009 in the Charité Universitätsmedizin Berlin, Berlin, Germany under third-party funds paid by EUROIMMUNE AG. CD and AR are employees of EUROIMMUN AG, Lübeck, Germany. WSchlumberger and WStöcker are board members of EUROIMMUN AG. The other authors have declared no conflict of interest.

## Authors' contributions

RB had full access to all of the data in the study and takes responsibility for the integrity of the data and the accuracy of the data analysis. RB, CD, FH and WSchlumberger contributed to study design. FB, RB, US, KE and AR contributed to the acquisition of data. FB, RB and FH contributed to analysis and interpretation of data. RB, GRB, WSchlumberger, CD and FH contributed to manuscript preparation. FB and RB contributed to statistical analysis. KE, CD, WStöcker, FH and WSchlumberger contributed to overall project management.
